# The Roles of Integrins in Function of Human Neutrophils after Their Migration through Endothelium into Interstitial Matrix

**DOI:** 10.1371/journal.pone.0118593

**Published:** 2015-02-23

**Authors:** Ding Luo, Helen M. McGettrick, Phil C. Stone, George E. Rainger, Gerard B. Nash

**Affiliations:** 1 School of Clinical and Experimental Medicine, College of Medical and Dental Sciences, University of Birmingham, Birmingham, United Kingdom; 2 School of Immunity and Infection, College of Medical and Dental Sciences, University of Birmingham, Birmingham, United Kingdom; Ludwig-Maximilians-Universität, GERMANY

## Abstract

We investigated the changes in neutrophil phenotype and function after transendothelial migration, and the roles played by integrin receptors in their behaviour. Neutrophils were tracked microscopically as they migrated through endothelial cells into collagen gels, and were retrieved at desired times. When endothelial cells were treated with increasing doses of tumour necrosis factor-α, neutrophils not only migrated in greater number, but also to a greater depth in the gel. Apoptosis was barely detectable in neutrophils retrieved after 24h, and many remained viable and motile at 48h. Neutrophils retrieved after 1h had increased oxidative capacity and at 24h had similar capacity as freshly-isolated neutrophils. However, by then they had impaired ability to phagocytose bacteria. Compared to fresh neutrophils, total mRNA was halved by 24h, but while β2-integrin expression decreased, β1- and β3-integrin increased along with ICAM-1. Studies of integrin blockade indicated that while β2-integrins were needed to cross the endothelial barrier, no integrins were required for migration within the gel. β2-integrins also contributed to phagocytosis, but their binding was not required for prolonged survival. These results demonstrate a model for integrated analysis of neutrophil migration and function, and describe development of effector functions and the roles of integrins in human neutrophils for the first time.

## Introduction

During inflammation, neutrophils are recruited from the blood stream and migrate through vascular endothelium to destroy pathogens and remodel damaged tissue. Subsequently the neutrophils undergo apoptosis and are themselves cleared by phagocytic macrophages to enable resolution of the inflammatory response [1]. The initial stages of adhesion from flow, and migration through endothelial monolayers have been studied and reviewed exhaustively [2,3], as have the abilities of neutrophils to release oxidants and proteolytic enzymes, and to phagocytose bacteria [4–6]. However, cell migration and functional responses have rarely been studied together or after neutrophils have crossed the endothelial barrier. Consequently, little is known as to how functions evolve during migration, and e.g., how well responses analysed using freshly isolated cells on two-dimensional surfaces represent those of cells which have migrated into the 3-D interstitial environment.

Integrated studies of the changes in neutrophil behaviour during and after transmigration have been hampered by the difficulty of following these processes in real time. Most *in vitro* studies have quantified neutrophil migration through endothelial monolayers alone (e.g., [7–9]). Occasionally following the transendothelial stage, migration under monolayers [10], through basement membrane [11] or on coated surfaces [12] have been analysed. In murine models, there have been detailed intravital studies of mechanisms controlling neutrophil migration through venular endothelium, underlying basement membrane and associated pericytes [13–16]. The above studies indicate that conditioning during transendothelial migration modifies neutrophil integrin usage and rate of migration under the endothelial cells (EC); while β2-integrins are required for adhesion to EC and migration through them, β1- and β3-integrins take on roles in the subendothelial space and crossing basement membrane [11,12,17]. Subsequent migration of neutrophils within interstitium directed by injected chemoattractants has been recorded [18–20]. Migration of leukocytes has also been studied *in vitro* within 3-D gels, which may be more representative of behaviour in tissues than migration on simple coated surfaces [21–23] although the cells have not been conditioned by migration through endothelium. Interestingly, studies examining migration of murine neutrophils and monocytes within tissue indicated that integrin-mediated adhesion was not required [24]. However, the role of integrins at the interstitial stage of migration after crossing endothelium has not been studied for human neutrophils.

Studies of the effector functions of migrated neutrophils, which may also be integrin-dependent, have also been held back by the lack of multi-stage, *in vitro*, 3-D models in which their sequential development could be followed. For freshly-isolated neutrophils at least, degranulation [25] and oxidant production [26] are amplified by integrin engagement, while phagocytosis is also integrin dependent [4,27]. However, functional studies over time are also made difficult because neutrophils are terminally differentiated and have a short life span, although this is believed to be prolonged once migrated into tissue [28]. Survival of neutrophils in culture can be prolonged by addition of various agonists such as granulocyte-macrophage colony stimulating factor, interleukin-8 or lipopolysaccharide [29–31]. We previously reported a marked delay in apoptosis for neutrophils migrating through endothelial monolayers that had been treated with tumour necrosis factor-α (TNF) [32], suggesting that functional studies would be possible over prolonged periods if migrated cells could be tracked or retrieved.

The foregoing raises questions regarding how the motility and effector functions of neutrophils change after they migrate across endothelium, how such changes are linked to changes in expression of integrins, and especially, whether integrins remain necessary for migration once cells have entered the interstitium. These questions could not be answered using existing in vitro experimental models available to study human cells, and so we developed a model in which EC were cultured on collagen gels and treated with TNF as a well-characterised initiator of neutrophil recruitment in other systems [33,34]. The model allowed us to visualize neutrophils migrating through the endothelial monolayer into an interstitial matrix, and also to retrieve them when desired. Thus we could study how motility changed with time after transendothelial migration and also use function blocking antibodies to test whether this motility was dependent on β1-, β2- or β3-integrins. We could also evaluate functional properties of retrieved cells and changes in their expression of integrins, to test how function and expression might be linked. We found apoptosis was greatly delayed in this milieu enabling prolonged studies of changes in neutrophil properties. Recovered neutrophils showed marked changes in integrin expression, oxidant production and phagocytic capability. Studies using inhibitors of integrin-mediated adhesion showed that while this was required for passage through endothelium into gels and phagocytosis, there was negligible requirement for such adhesion for later migration or survival. Thus, we report a unique model for studying neutrophil migration and function for prolonged periods in an integrated fashion, and describe for the first time the role of integrins in function of human neutrophils after transendothelial migration.

## Materials and Methods

### Isolation of human neutrophils

Venous blood from healthy adult volunteers was collected in EDTA tubes (Sarstedt, Leicester, UK). Neutrophils were isolated by centrifuging whole blood over a two-step density gradient as previously described [35], washed twice in PBSA (phosphate-buffered saline containing 0.15% bovine serum albumin; BSA, cultured tested solution; Sigma-Aldrich Co, UK) and suspended in Medium-199 (M199, Gibco Invitrogen Compounds, Paisley, Scotland) supplemented with 0.15% BSA (M199/BSA) at 10^6^/ml. EDTA was chosen as anticoagulant as in our experience, it provides a high yield of cells with low level of activation judged by retention of spherical shape [36].

### Culture of EC on collagen gels

To form collagen gels, rat tail collagen type I (First Link Ltd., West Midlands, UK), was mixed with 10X M199 (Sigma) and subsequently neutralised with 1N NaOH to yield a final concentration of 1.8mg/ml [37]. Gels were dispensed into 6-well plates, allowed to set at 37°C and then equilibrated with 1X M199 for 48h.

Human umbilical vein endothelial cells (HUVEC) were isolated and cultured in medium M199 supplemented with 20% heat-inactivated fetal calf serum, 2.5μg/ml amphotericin B, 1μg/ml hydrocortisone, 10ng/ml epidermal growth factor (all from Sigma) and 35μg/ml gentamycin (Gibco) until confluent, as previously described [38]. Primary HUVEC were dissociated using trypsin/EDTA (Sigma) and seeded onto the collagen gels at a density chosen to yield confluent monolayers within 2 days. TNF (0, 1, 10 or 100U/ml; Sigma) was added to confluent monolayers for 4 hours before assay.

### Ethics statement

The study was carried out after ethical approval from the Science, Technology, Engineering and Mathematical Ethical Review Committee of the University of Birmingham. Blood donors were healthy adult volunteers who gave written informed consent. Capacity to consent was considered to be established by their status as members of staff or students of the University of Birmingham. Consent forms were of a format and were filed in accordance with procedures laid down by that committee. Human umbilical cords were obtained from the Human Biomaterials Resource Centre (University of Birmingham) after informed consent.

### Neutrophil migration through EC into collagen gels

All manipulations of gels and microscopy were carried out at 37°C. HUVEC were washed with M199/BSA to remove residual TNF. Neutrophils (2ml at10^6^/ml) were added and after 10 min settling, the non-adherent cells were removed by washing. The cells were observed using a phase-contrast microscope with motorised Z-focus and digital camera under computer control using Image-Pro Plus software (Media Cybernetics, UK). Digitised Z-stack images were acquired at 2μm steps through the depth of the gel starting at the EC surface in 5 random fields at 15min, 1, 3 and 24h, and analysed offline using the same software. Neutrophils were counted as they came into focus, averaged for the five fields and converted to cells/mm^2^ using the calibrated field dimensions. This number was multiplied by the known surface area of the HUVEC, and divided by the known number of neutrophils added to obtain the percentage of neutrophils that had adhered. At the endothelial monolayer, neutrophils were divided into those that were phase-bright (above EC) and those that were phase-dark (migrated just below EC). The percentages of neutrophils that had (i) transmigrated (sum of phase-dark cells and those that had entered the gel) or (ii) that had entered the gel were calculated. Finally, the depths to which transmigrated neutrophils had penetrated the gels were analysed. The digital Z-stack was played back from the top to the bottom of the gel (see e.g., S1 Video); the position of the phase-dark cells just under the endothelium was assigned zero and each subsequent image was 2μm further down; the total depth was 500μm. Neutrophils were counted as they came into focus, and those counted in the first 50μm were assigned the depth 25μm, those in the second 50μm were assigned the depth 75μm, and so on. The process was repeated for the 5 different fields recorded. The average depth of penetration was then calculated for all the cells.

In some experiments, a single field approximately half-way into the gel was digitally recorded for 10 min to analyze neutrophil motility (see e.g., S2 Video). The percentage of cells that were migrating was calculated from the number of cells that were mobile and the number stationary (displacement less than one cell diameter). The migration velocity of a cell was assessed by using the Image-Pro software to allocate its x-y co-ordinate in 11 digitised images recorded one minute apart. The average movement in the x-y plane per minute was calculated, and this migration velocity was evaluated for 10 cells. The value characterises a 2-D projection of 3-D motion. If one assumes a random direction of migration, it can be multiplied by (1.5)^½^ to obtain the 3-D 'total' velocity.

### Recovery of neutrophils from gel

Neutrophils were recovered from the gel at chosen times for analysis. HUVEC were dissociated with 2mg/ml dispase II (Sigma) at 37°C until the monolayer had detached. The surface of the gel was washed 3 times with PBS. Under these conditions, neutrophils adherent to the monolayer and migrated just below it were removed. The gel was then digested in collagenase III (Sigma #C0255) at a final concentration of 125U/ml at 37°C for 30 min. Recovered cells were washed twice and resuspended in PBSA. This collagenase III was chosen as we found that it had minimal effect on neutrophil activation as assessed by morphological changes when freshly-isolated cells were incubated with 125U/ml for 30 min at 37°C. In addition, we tested the effect of collagenase III on each of the neutrophil functional assays (see below), by pre-treating cells with the enzyme for the same period.

### Neutrophil migration into collagen gels without endothelial monolayers

In some experiments, migration of freshly isolated neutrophils into gels containing formyl-methionyl-leucyl-phenylalanine (fMLP), without an endothelial monolayer was studied. Collagen gels were made as above (800 μl in 24-well plates) and M199 with fMLP (0 or 2 x 10^–7^ M) was added for 24h to allow diffusion of fMLP into the gel. This was replaced with fresh M199 30min prior to experiment to create a fMLP gradient. Medium was removed and neutrophils were added (1.25 x 10^5^/ml in 800μl) and migration into the gel was assessed at 30 and 60min, as described above. The proportion of added cells entering the gel, and their average depth were analysed. In separate experiments, after allowing neutrophils to settle for 10min, non-adherent cells were washed off the top of the gel and counted, to obtain an independent measure of the proportion of neutrophils initially adhering to gels.

### Apoptosis

Retrieved neutrophils were centrifuged onto glass slides in a Shandon Cytospin II (Scientific Instruments, South Trentham, UK) fixed with methanol and stained with Diff Quik (Dade Behring Ltd, UK). At least 100 cells were counted using a 60x oil-immersion lens, with apoptotic cells defined as containing one or more darkly stained condensed nuclear fragments [39]. Alternatively, retrieved neutrophils were fixed and permeabilised (Invitrogen Fix & Perm kit, GAS-004) and incubated with rabbit anti-human active caspase 3 or isotype matched control IgG (both 6μg/ml; BD Pharmingen), followed by phycoerythrin-conjugated goat-anti-rabbit (5μg/ml; Southern Biotech). Fluorescence intensity of neutrophils was measured in a FACScan flow cytometer (Becton Dickinson Ltd, Oxford, UK). Median fluorescence intensity (MFI) was calculated after subtraction of the value for control IgG, and positive cells were calculated as the percentage with intensity greater than 97% of the cells labelled using control IgG. The retrieved cells were compared to freshly isolated neutrophils, and neutrophils cultured in M199/BSA in a 6-well plate at 37°C for 24 or 48h. In addition, in separate experiments, neutrophils were cultured in M199/BSA at 37°C for 24 or 48h on unstimulated EC that had been cultured to confluence in 6-well plates.

### Oxidant production

Neutrophil oxidant production was quantified by the conversion of non-fluorescent dihydrorhodamine 123 (DHR123) into fluorescent rhodamine 123. Neutrophils recovered from gels or freshly isolated cells were warmed to 37°C for 5 min in 1ml PBSA, incubated with 2μl of 500μM DHR123 (Invitrogen) for 5min, and incubated for a further 15min with or without 1μM fMLP. To stop the reaction, cells were washed in ice-cold PBSA and fixed with ice-cold 2% formaldehyde. MFI of neutrophils was measured as above, with intensity for non-stained cells subtracted.

Neutrophil superoxide production was measured by the superoxide dismutase (SOD) inhibitable reduction of ferricytochrome C. Neutrophils were primed by pre-incubating with TNF (100 U/ml) and cytochalasin B (5μg/ml) at 37°C for 15min. In a 96-well plate, neutrophils (50 μl of 2 x 10^6^ /ml) were added to 180μl PBS, 10μl cytochrome C (23mg/ml; Sigma), and in some wells 10μl SOD (30,000U/ml; Sigma). Finally, 1μM fMLP was added to chosen wells. The plate was incubated at 37°C and the optical density (O.D.) was read at 550nm at time zero and then at intervals for up to 120min. Superoxide production was calculated using a molar extinction coefficient for ferricytochrome c (21.1 × 10^3^ M^−1^ cm^−1^) and a light path of 0.6 cm for a final well volume of 0.25ml.

### Phagocytosis

Neutrophils (2 x 10^5^ in 100 μl) were mixed with un-opsonised pHrodo *E. coli* BioParticles Conjugates (100μl at 1mg/ml in PBS with 20mM HEPES, pH 7.4; Invitrogen,) and incubated serum-free at 37°C for 10, 30min or 1h. Samples were then fixed with 2% formaldehyde with 20mM HEPES (pH 7.4), washed and resuspended in PBS (with 20mM HEPES, pH 7,4) before imaging using a confocal microscope or analysis of MFI by flow cytometer. The particles have a low fluorescence intensity in standard media or when adhered to the surface of neutrophils, but increase their fluorescence when taken into acidic phagocytic vacuoles.

### Inhibition of integrin function

The following antibodies were used at 10μg/ml to block integrin function: rat IgG2a anti-human CD29/β1-integrin (Mab13; BD Pharmingen); mouse IgG2a anti-human CD18/β2-integrin (IB4; Calbiochem); mouse IgG1 anti-human CD61/β3-integrin (SZ21; Beckman Coulter); matched control antibodies, rat IgG2a, mouse IgG2a (both eBioscience), mouse IgG1 (DAKO). In some experiments, RGDS peptide (Arg-Gly-Asp-Ser, 0.5 mM; Sigma) or CT7010 (20 μM) was used instead of antibody. CT7010 is a low molecular weight, non-peptide, inhibitor of β_2_-integrin function (gift of Dr. Tony Shock, Celltech R&D) The above antibodies and inhibitors have previously been shown to block functions [11,40,41].

Neutrophils were treated with antibodies for 10 min prior to addition to the gels. For endothelial monolayers, after 10 min the non-adherent cells were removed by washing with M199/BSA (0.15%) and 1 ml of M199 BSA (0.15%) containing the agent was re-added to the gel. In separate experiments, agents were added after 15min when initial migration had occurred. We showed that these added antibodies did reach and bind to integrins within 1h by retrieving cells, labelling them with secondary anti-integrin and flow cytometry (see below). Neutrophils were also treated with antibodies 10min before analysis in the phagocytosis assays.

### Expression of integrins and of ICAM-1 at mRNA and protein levels

Quantitative real time PCR (qPCR) was carried out to evaluate gene expression. For retrieved neutrophils, any residual HUVEC were removed using a Mo Flo high speed cell sorter (Beckman-Coulter; Dako) and positive selection of neutrophils which had been labelled with antibody against CD11b/α_m_-integrin (Dako R0841, 1:100); sorted purity was ≥ 99% neutrophils. RNA was extracted from known numbers of neutrophils using TRIzol (Invitrogen) and purity and quantity of mRNA was analysed using a NanoDrop ND-1000 full-spectrum (220–750nm) spectrophotometer (NanoDrop products, Wilmington, DE). RT-PCR was conducted with a QuantiTect probe RT-PCR kit (Qiagen) following the manufacturer’s instructions. β1-, β2-, β3-integrin and ICAM-1 FAM-labelled primers were brought as Assay on Demand kits from Applied Biosystems (Warrington, UK). VIC-labelled 18S (Eurogenetec, Southampton, UK) was used as a housekeeping gene. Samples were amplified using a model 7900HT real-time PCR machine and analyzed in SDS2.2.2 (Applied Biosystems). Data were initially expressed as relative quantification (RQ) relative to 18S, before comparison between treatments.

To assess surface protein expression, retrieved or freshly-isolated neutrophils were incubated on ice for 30min with one of the following murine mAb; FITC-conjugated anti-ICAM-1 (10μg/ml; Caltag), anti-β1 (1/50; Abcam) or anti-β2-integrin (1/50; R&D); PE-conjugated anti-β3-integrin (1/50; R&D); isotype-matched control antibodies FITC-IgG2a (R&D), FITC-IgG1 (DAKO), PE-IgG2a (R&D). In some experiment, when anti-β2-integrin (IB-4) was added after addition of neutrophils to the endothelial monolayer, the neutrophils were retrieved from the gel and incubated for 30min with goat- anti-mouse IgG-FITC (1/50; DAKO), to test whether the antibody had reached all cells. Cells were washed in ice cold PBSA (2% BSA) and fixed in 2% formaldehyde for 15min. MFI of neutrophils was measured as above, with intensity for isotype-matched control subtracted.

### Statistical analysis

Variation between multiple treatments and between multiple time points was evaluated using analysis of variance (ANOVA). In cases showing significant variation, post hoc comparisons were made to control by Dunnett test or between conditions by Bonferroni. When treatments were compared over a range of time points, post hoc comparisons of treatments used combined time points. Effects of single treatments were analysed by paired t test compared to controls.

## Results

### Kinetics of neutrophil migration through endothelial cells into collagen gels

**Fig 1:**
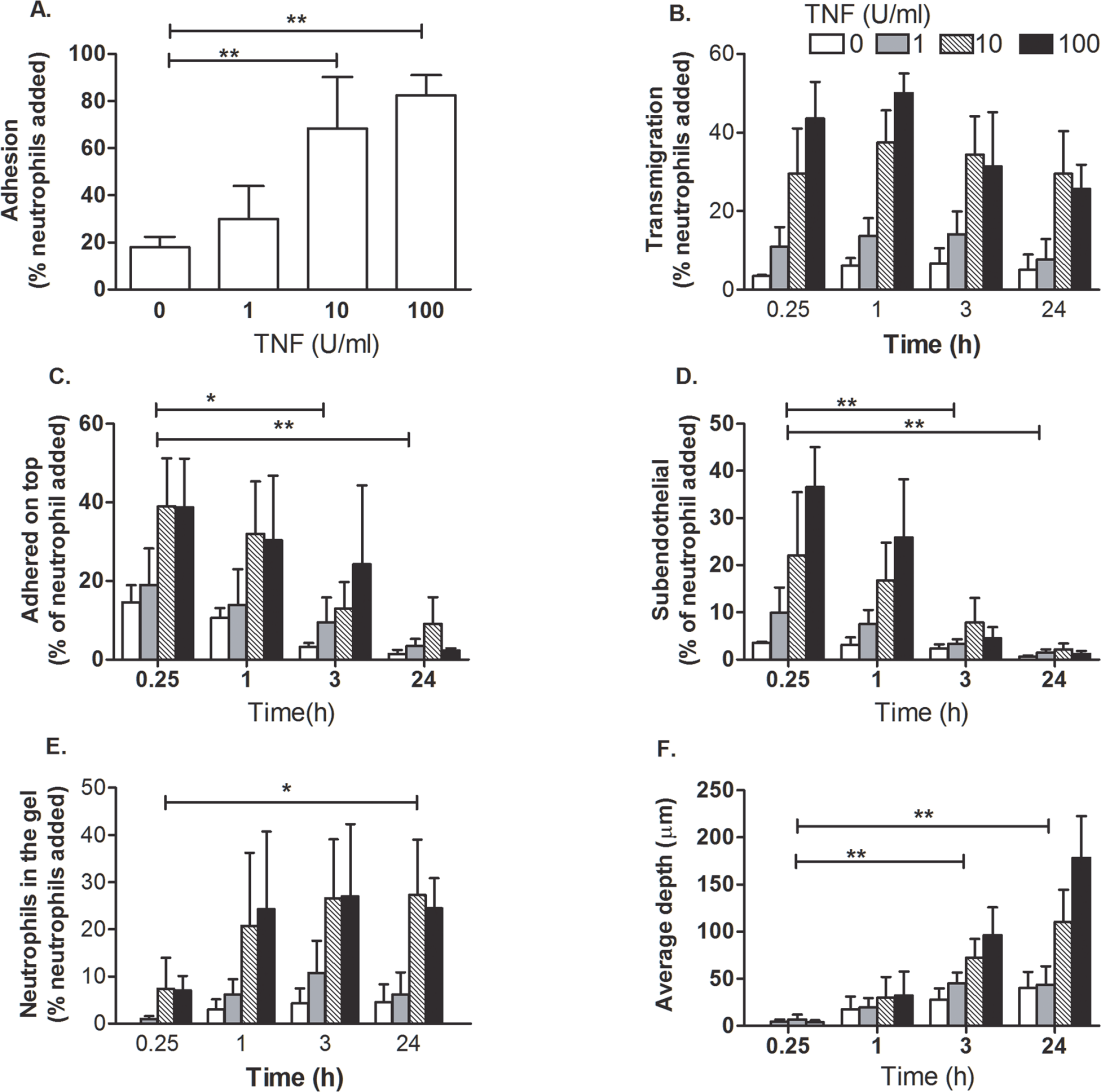
Effect of TNF concentration on neutrophil adhesion and migration through EC and into collagen gels. HUVEC were stimulated with 0, 1, 10 or 100U/ml TNF for 4 h. Neutrophils were added and allowed to adhere for 10 min before non-adherent cells were washed off and data collected at 15 min, 1 h, 3 h and 24 h. A. Total adherent neutrophils after 15min (% of those added). B. Neutrophils that were transmigrated across the EC (% of those added). C. Neutrophils that were adherent on top of the endothelial monolayer (% of those added). D. Neutrophils that had transmigrated across the EC but were just under the monolayer (% of those added). E. Neutrophils that had migrated into the gel (% of those added). F. Average depth penetrated by the neutrophils that had transmigrated. In A and B, ANOVA showed a significant effect of TNF concentration (p<0.05 and p< 0.01 respectively). In C-F, ANOVA showed a significant effect of time and TNF dose (p<0.01 in every case). * = p<0.05, ** = p<0.01 compared to the initial time point by Dunnett test. Data are mean ± SEM from 3 experiments.

We examined neutrophil adhesion, transmigration and penetration into the collagen gel after stimulating HUVEC with 0, 1, 10 or 100 U/ml TNF. The proportion of neutrophils initially adhering and the proportion of neutrophils that transmigrated (i.e., all cells found below the endothelial layer) increased with increasing dose of TNF (Fig. 1A,B), with few cells recruited 0 or 1U/ml. Regarding time, total transmigration increased rapidly within 15 min and reached a maximal level by 1h, but subsequently decreased over 3–24h (Fig. 1B). The later decrease was attributed to 'reverse migration' by neutrophils which we observed previously for endothelial monolayers cultured without underlying gels [42]. The number of the cells on the top surface of the endothelial cells decreased steadily with time (Fig. 1C), initially because cells were migrating into the subendothelial space, and then during reverse migration, because the reverse migrated and residual surface cells were gradually removed when the surface was washed at each time point. Of the transmigrated cells, a proportion were phase dark cells just under the endothelial monolayer, and a proportion migrated into the gel. The former decreased with time (Fig. 1D) while the latter increased (Fig. 1E). Thus, after 1h, transmigrated cells either moved further in the gel or reversed migrated above the endothelium, with few remaining just below the monolayer. In consequence, for TNF treatments of 10 and 100U/ml at 1,3 or 24h (conditions when cells were retrieved from the gel for analysis in subsequent experiments), of the cells that were transmigrated, ∼50%, 25% or 10% were on average just below the EC respectively. Finally, neutrophils penetrated deeper in the gel over time, but also with increasing dose of TNF (Fig. 1F). Thus, the concentration of TNF affected adhesion and transendothelial migration as expected from our previous studies [33] but also influenced the efficiency and depth of penetration of the gel.

### Neutrophil survival and motility after transendothelial migration

**Fig 2:**
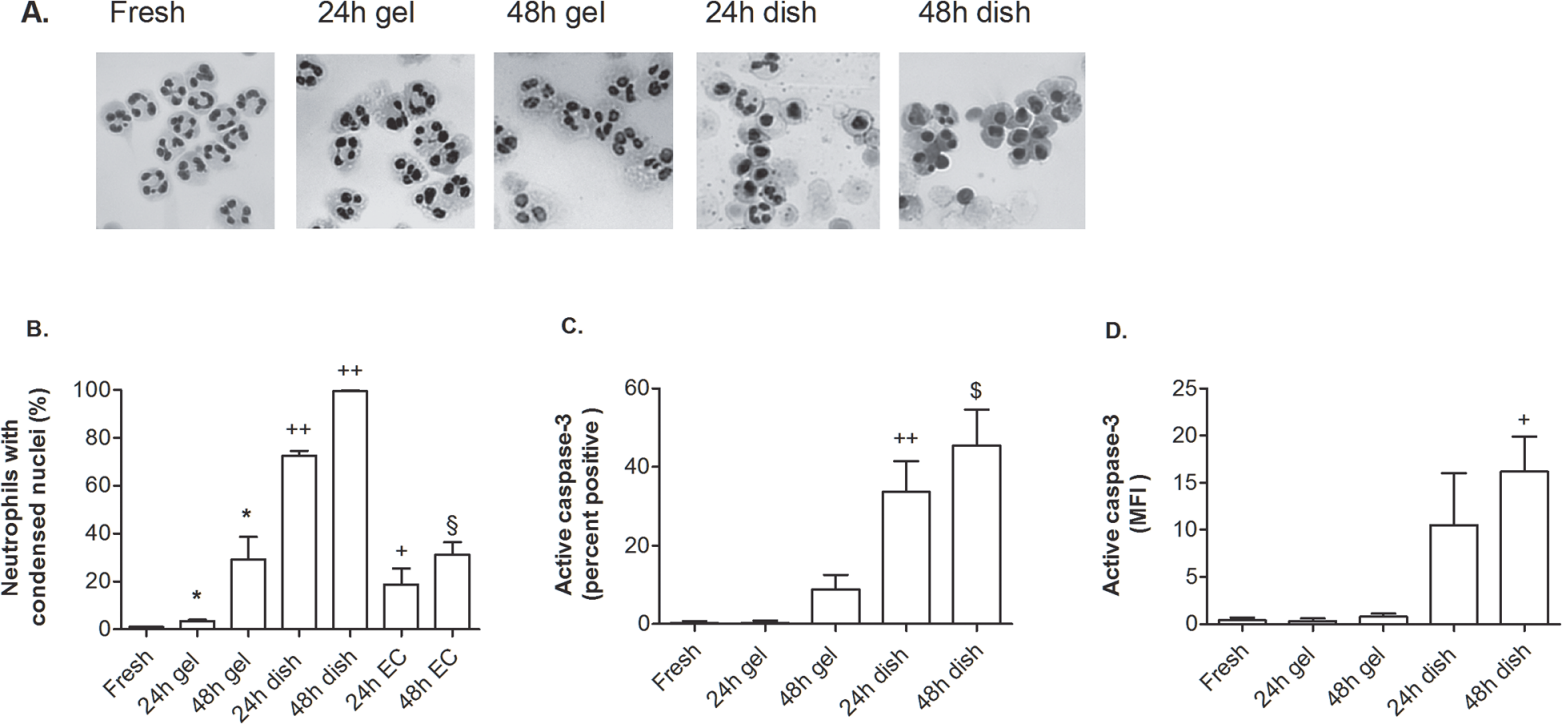
Effects of migration and culture on apoptosis of neutrophils. Freshly isolated cells (Fresh) were compared to: cells cultured 24 or 48 h in a collagen gel having migrated through EC treated with 100U/ml (24h gel, 48h gel); cells cultured 24 or 48 h in a dish (24h dish, 48h dish); cells cultured on top of unstimulated EC in a dish (24h EC, 48h EC). A. Microscopic images showing nuclear morphology for freshly isolated neutrophils; those retrieved from the gel 24 or 48h after migrating through TNF-treated endothelial cells; those incubated in a plastic dish for 24 or 48h. B. The percentage of neutrophils that had condensed nuclei under the same conditions, and also for neutrophils that were incubated on unstimulated endothelial monolayers for 24 or 48h. C. Percentage of neutrophils positive for active caspase 3 by flow cytometry for freshly isolated neutrophils; those retrieved from the gel 24 or 48h after migrating through TNF-treated endothelial cells; those incubated in a plastic dish for 24 or 48h. D. MFI of neutrophils labelled for active caspase 3 by flow cytometry for freshly isolated neutrophils; those retrieved from the gel 24 or 48h after migrating through TNF-treated endothelial cells; those incubated in a plastic dish for 24 or 48h. Data are mean ± SEM from 4 or 5 experiments. * = p<0.05 compared to freshly isolated cells by paired t test. $ = p = 0.051, + = p<0.05, ++ = p<0.01 compared to neutrophils in gels after the same time period by paired t test. § = p<0.05, compared to neutrophils in gels by unpaired t test.

When cultured in a plastic dish, a large proportion of neutrophils underwent apoptosis by 24h and nearly all by 48h, as assessed by condensed nuclei (Fig. 2A,B). In contrast, migration through EC treated with 100U/ml TNF into collagen gels protected neutrophils from undergoing apoptosis, with little change in nuclear morphology at 24h and slightly more at 48h (Fig. 2A,B). For comparison, neutrophils were also incubated on unstimulated EC monolayers; they also showed quite low levels of nuclear condensation, with values higher than cells in gels at 24h but similar at 48h (Fig. 2B). We also assessed active caspase-3 in neutrophils and found negligible levels for fresh neutrophils and much higher levels for cells cultured in dishes (Fig. 2C,D). The cells retrieved from gels showed little activation at 24h and slightly more at 48h (Fig. 2C,D). When neutrophils migrated through EC stimulated with a lower dose of TNF (10U/ml), only 6% had condensed nuclei at 24h (mean from 2 experiments).

Directly observing transmigrated neutrophils within the gel 24 h after addition to EC treated with 100U/ml TNF, 75±5% were motile and their average 2-D projected velocity was 4.9 ± 0.5 μm/min (mean ± SEM from three experiments; see S2 Video). The percentage of motile neutrophils dropped to 35% after 48 h (mean of 2 experiments; see S3 Video), but ones that were moving averaged essentially the same velocity (5.1 μm/min). Again, similar results were seen when neutrophils migrated through EC treated with 10U/ml TNF at 24h (50± 6% motile, velocity 3.7± 0.1 μm/min; mean ± SEM; n = 3).

### Neutrophil function after transendothelial migration

**Fig 3:**
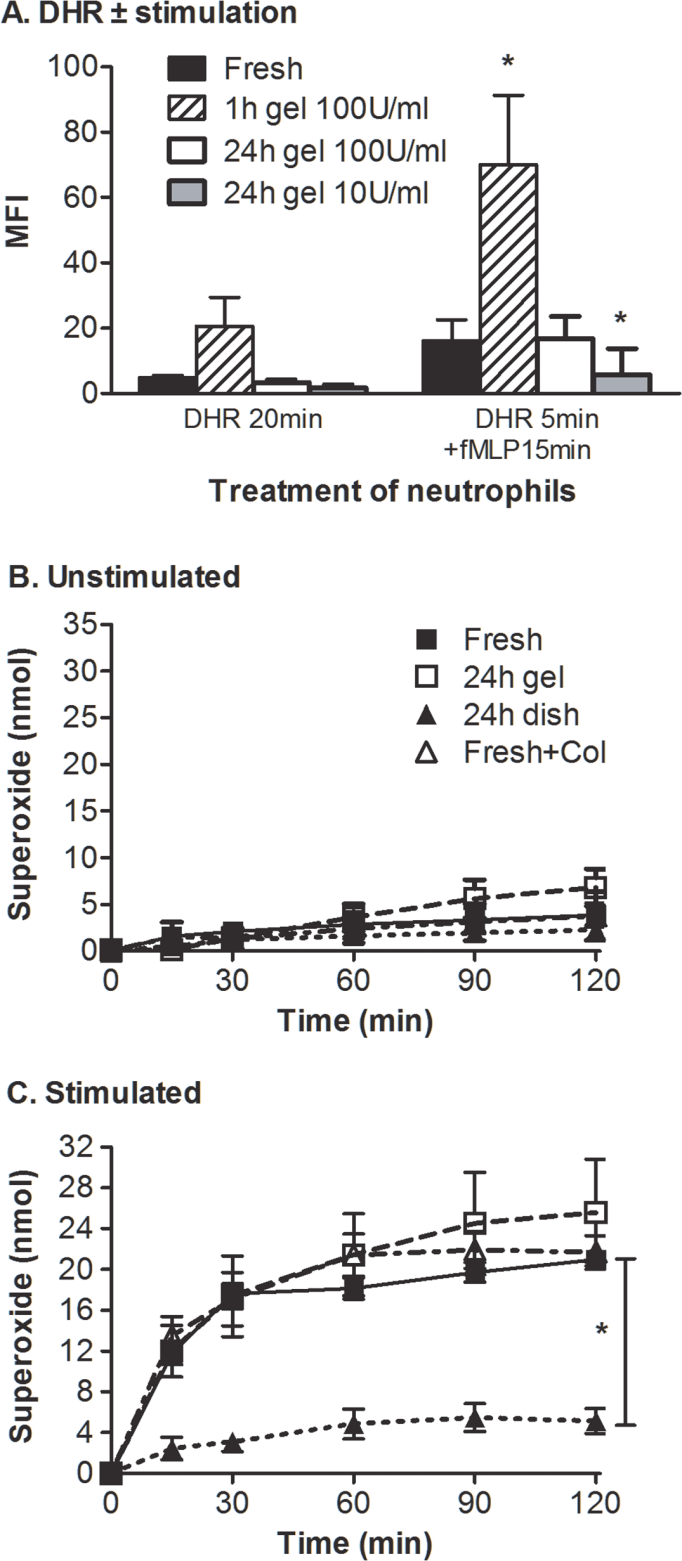
Effects of migration and culture on oxidant production by neutrophils. A. Oxidant production by neutrophils with or without stimulation with fMLP assayed by measuring MFI of cells loaded with DHR123. Freshly isolated cells were compared to cells cultured 1 h or 24 h in a collagen gel having migrated through EC treated with 100U/ml or 10U/ml TNF. Data are mean ± SEM from 4 experiments. ANOVA showed a significant effect of gel entry (p<0.05); * = p<0.05 compared to Fresh by Bonferroni test. B,C. Superoxide production by neutrophils either unstimulated (B) or treated with fMLP (C). Neutrophils were freshly isolated, cultured 24h in a collagen gel having migrated through EC treated with 100U/ml TNF (24h gel), cultured 24h in a plastic dish (24h dish) or freshly isolated but treated with collagenase for 30min. Data are mean ± SEM from 3 experiments. In D, ANOVA showed a significant effect of treatment (p<0.05) and time (p<0.01); post-hoc analysis showed that cells cultured in a dish, but not those in a gel, were significantly different from freshly isolated cells; * = p<0.05 compared to Fresh by Dunnett test.

**Oxidant production.** Having established that migrated neutrophils survived for prolonged periods, we analysed their effector functions. Oxidant production was tested using two complementary methods which assess different products: (i) conversion of DHR123 into fluorescent rhodamine 123 which is mediated by intracellular hydrogen peroxide, and allows analysis of relative production for a population of individual cells by flow cytometry; (ii) reduction of ferricytochrome C assayed colorimetrically which gives an absolute measure of superoxide released into the extracellular space. In each method, basal production is very low and the bacterial formyl peptide fMLP was added to test the response of neutrophils upon stimulation. For DHR123, neutrophils recovered from gels 1h after migrating through EC (100U/ml TNF) showed slightly (but not significantly) increased basal conversion of the dye, but upon stimulation showed significant, several-fold greater conversion compared to freshly isolated cells (Fig. 3A). This capability was reduced by 24h and no longer significantly different from freshly isolated cells, while neutrophils retrieved 24h after migration through EC treated with 10U/ml TNF had lower oxidant production than freshly isolated neutrophils (Fig. 3A).

As expected, freshly isolated cells and variously incubated cells all produced similar low levels of superoxide without stimulation (Fig. 3B). Upon stimulation with fMLP, neutrophils retrieved 24h after migration through EC treated with 100U/ml TNF showed a slightly, but not statistically significantly, higher level of superoxide production compared to freshly isolated neutrophils (Fig. 3C). Treatment of freshly isolated cells with collagenase did not modify oxidant production, but cells incubated in plastic dishes for 24h showed much reduced response to fMLP compared to fresh cells (Fig. 3C).

### Phagocytosis

**Fig 4:**
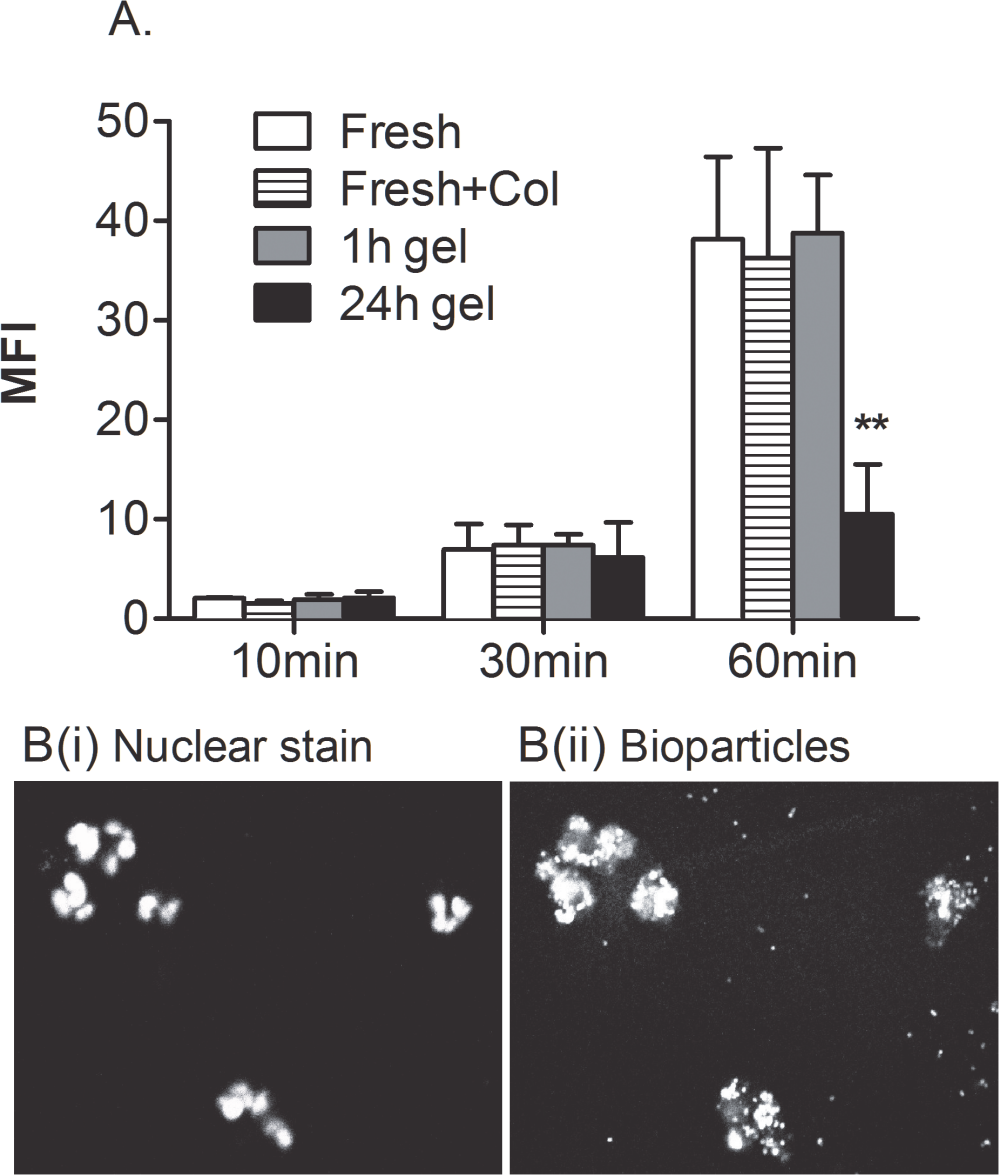
Effects of migration and culture on phagocytosis by neutrophils. Neutrophil phagocytosis was analysed by measuring intensity of fluorescence of cells incubated with pHrodo *E. coli* BioParticles. Freshly isolated neutrophils (without or with collagenase treatment) were compared to neutrophils recovered from a gel 1h or 24 h after migrating through EC treated with 100U/ml TNF. A. Samples were incubated with bioparticles for 10, 30 or 60min before being fixed for flow cytometry. B. Confocal microscopic images of neutrophils recovered after 1h and incubated with bioparticles for 60min, imaged in the same plane for (i) bisbenzamide-stained nuclei or (ii) fluorescent intracellular bioparticles. Data are mean ± SEM from 3 experiments. ** = p<0.01compared to freshly-isolated cells by paired t test.

We also tested the ability of migrated neutrophils to phagocytose *E. coli* particles. After incubating freshly-isolated neutrophils with *E. coli* bio-particles for 30 min, we detected a small increase of fluorescence. By 60 min, a further 5-fold increase in fluorescence intensity was seen (Fig. 4A). Confocal microscopy of similarly-treated cells indicated that fluorescent bacteria were internalised at 60 min (Fig. 4B). Collagenase-treated, freshly-isolated neutrophils gave similar signals, as did migrated neutrophils extracted from gel after 1h (Fig. 4A). However, neutrophils retrieved 24 h after migration showed a much smaller increase in fluorescence after 60 min, although they did show the initial rise in fluorescence at 30 min (Fig. 4A). This suggests that these cells bound the bacteria but failed to take them up.

### Changes in gene and protein expression after transendothelial migration

**Fig 5:**
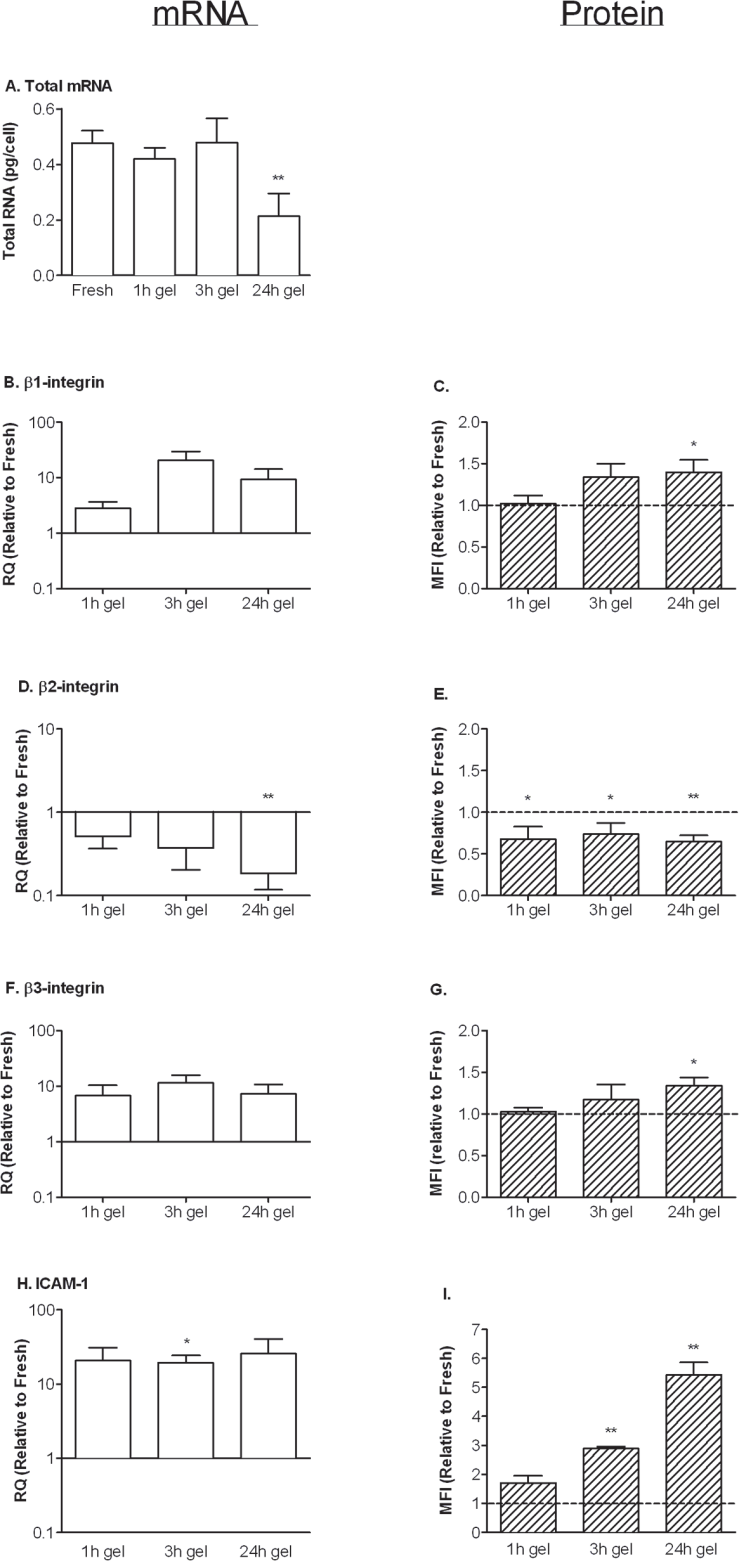
Effects of migration and culture on gene and surface protein expression by neutrophils. A. Total mRNA extracted from neutrophils (pg/cell). Freshly isolated neutrophils (Fresh) were compared to neutrophils recovered from a gel 1h, 3h or 24h after migrating through EC treated with 100U/ml TNF. B,C. mRNA and surface protein expression of β1-integrin. D,E. mRNA and surface protein expression of β2-integrin. F,G. mRNA and surface protein expression of β3-integrin. H,I. mRNA and surface protein expression of ICAM-1. In B-I, values for neutrophils recovered from a gel at 1 h, 3 h or 24 h, are expressed relative to values for freshly isolated cells. Data are mean ± SEM from 3 to 10 independent experiments. * = p<0.05, ** = p<0.01 compared to freshly-isolated cells by paired t test.

Having characterised kinetics of migration and changes in apoptosis and behaviour of neutrophils during the period after migration, we aimed to evaluate the roles of integrins in these phenomena. We started by evaluating whether expression of β1-, β2- or β3-integrins changed with time at the mRNA levels. mRNA was extracted from known numbers of cells recovered from the gels and total mRNA per cell was quantified to evaluate total transcriptional activity, and also assist interpretation of the values for relative expression of the individual genes. Total mRNA per migrated cell retrieved after 1 or 3h was similar to freshly isolated cells, but after 24h this was significantly reduced by half (Fig. 5A). qPCR showed up-regulation of mRNA for β1- andβ3-integrin and (Fig. 5B,F) and down-regulation of β2-integrin (Fig. 5D) relative to 18S. The changes were consistent in trend over time but quite variable in magnitude, so that only some time points showed statistical significance. Combined with the data for absolute quantities of mRNA in cells, the results indicate that β1- and β3-integrins were still up regulated at 24h, when β2-integrins had dropped to a very low level in absolute terms. Changes in surface expression of integrins measured by flow cytometry showed similar trends to mRNA qualitatively. There was a progressive increase in surface expression of β1- and β3-integrins (Fig. 5C,G) and decrease in β2-integrin (Fig. 5E) which were all statistically significant by 24h, reaching 30–40% change in each case. We also evaluated changes in expression of ICAM-1 as a reference marker found to increase in migrated neutrophils in vivo (55,56). Indeed, ICAM-1 was markedly up regulated at the mRNA level in cells retrieved from the gel (Fig. 5H) and progressively in surface expression (Fig. 5L).

### Role of integrins in neutrophil adhesion and migration

**Fig 6:**
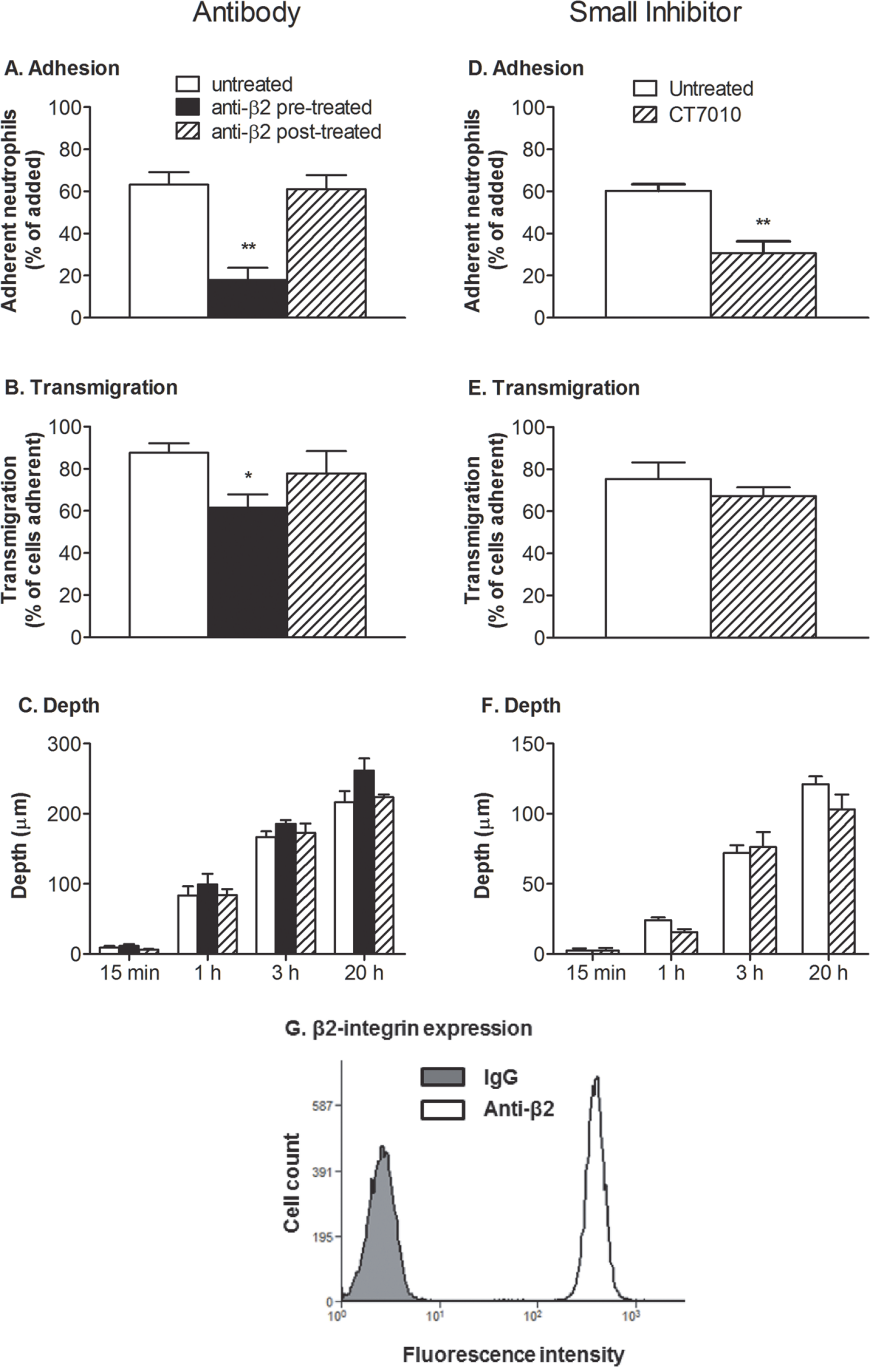
Effects of inhibition of β2-integrin function on adhesion to TNF-treated EC and migration into subendothelial matrix. HUVEC were stimulated with 100U/ml TNF for 4 h and neutrophils were added and allowed to adhere for 10 min before non-adherent cells were washed off and data collected at 15 min, 1 h, 3 h and 24 h. A,D: Total adherent neutrophils after 15min (% of those added). B,E: Neutrophil transmigration after 1 hour (% of those adherent). C,F: Average depth penetrated by the neutrophils that had transmigrated. In A-C neutrophils were untreated, pre-treated with antibody against β2-integrin, or antibody was added after 15min. InD-F, neutrophils were untreated, or gels were loaded with a small molecule inhibitor of β2-integrin (CT7010). G: Fluorescence intensity distribution for neutrophils retrieved from the gel at 3h when antibody against β2-integrin was added after 15min, assessed by flow cytometry. In A and B, ANOVA showed a significant effect of treatment (p<0.01 in each case); * = p<0.05, ** = p<0.01 by Dunnett test compared to untreated. In D, ** = p<0.01 by paired t-test compared to untreated. In C and F, ANOVA showed significant effect of time (p<0.01 in each case) but not treatment. Data are mean ± SEM from 4 experiments (A-C) or 5 experiments (D-F).

Having shown that integrin expression changed after transmigration, we proceeded to test which of the integrins might be required to allow migration within the gel. Function-blocking agents were used to inhibit the adhesive function of each family of integrins for cells migrating through EC treated with 100U/ml TNF. Pre-treatment of neutrophils with antibody against β2-integrin markedly reduced neutrophil adhesion (∼70%) as expected (Fig. 6A). Of those cells that were adherent cells, there was a smaller (∼30%) but still significant reduction in the percentage that transmigrated (Fig. 6B). To avoid the effect on initial attachment, antibody was also added 10 min after neutrophils had settled, which did not reduce adhesion (Fig. 6A) or the percentage that transmigrated (Fig. 6B) significantly. Those pre-treated neutrophils that did transmigrate actually migrated slightly further into the gel, but when antibody was added after 10min, the distance travelled into the gel was not altered compared to control (Fig. 6C). It is important that antibody blockade persists throughout the assay, and so we analysed binding by flow cytometry. We verified that 100% of the neutrophils were positively labelled with antibody added after initial adhesion when they were retrieved from the gel after 3h, showing that antibody had effectively bound all cells (Fig. 6G). We also incorporated a small molecule inhibitor CT7010 in the gel at the time of its formation, so that it was present throughout the assay. Because diffusion out of the gel would occur and dilute the agent at each medium change, we used 20μM when previous studies showed inhibition of β2-integrin at 1μM [32]. We found that in the presence of the inhibitor, adhesion was about 50% untreated control (Fig. 6D), transmigration was only slightly reduced (Fig. 6E), but that the depth of travel was not significantly modified (Fig. 6F).

**Fig 7:**
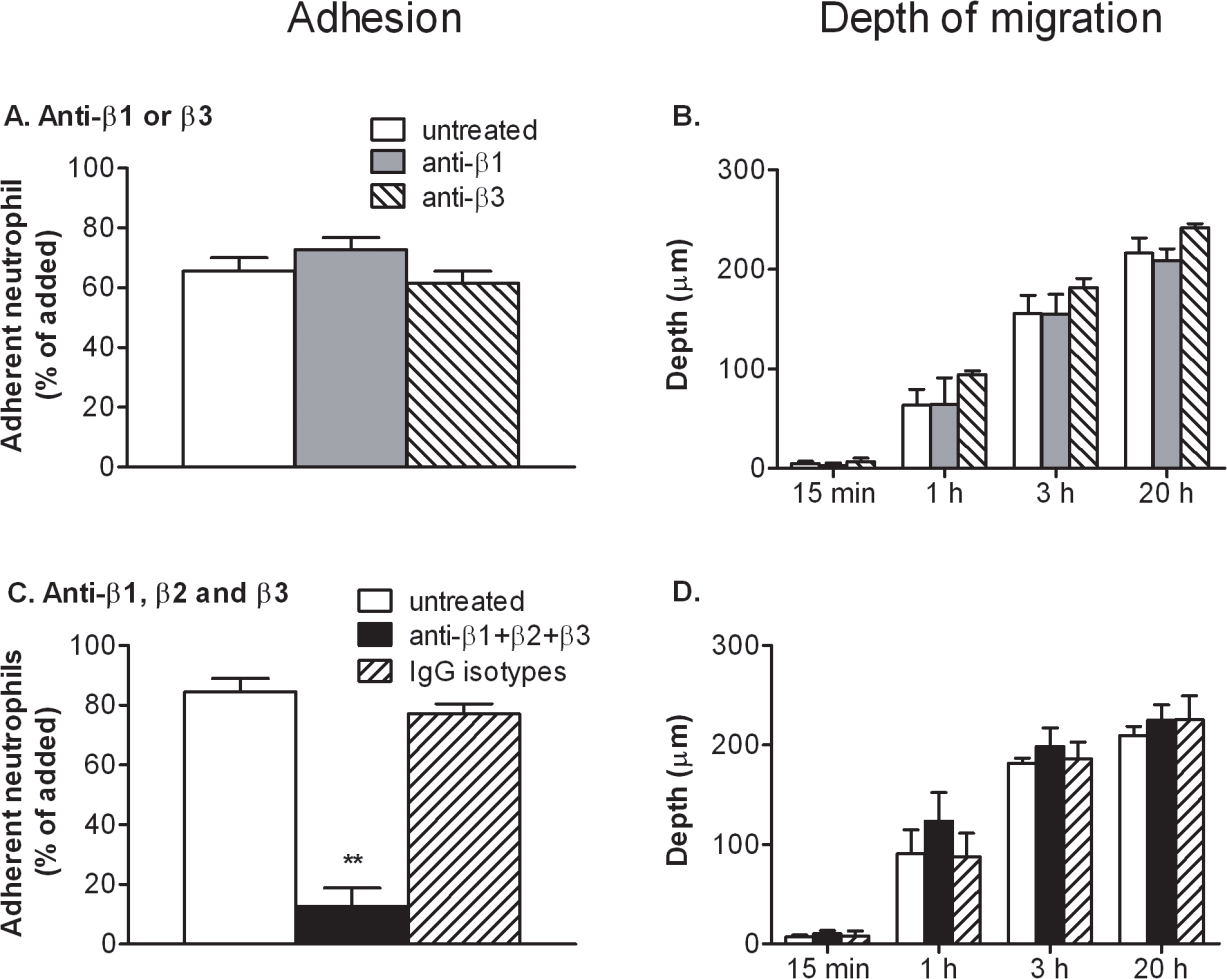
Effects of inhibition of integrin function on adhesion to TNF-treated EC and migration into subendothelial matrix. HUVEC were stimulated with 100U/ml TNF for 4 h and neutrophils were added and allowed to adhere for 10 min before non-adherent cells were washed off and data collected at 15 min, 1 h, 3 h and 24 h. A,C: Total adherent neutrophils after 15min (% of those added). B,D: Average depth penetrated by the neutrophils that had transmigrated. In A, B, neutrophils were untreated, or pre-treated with antibody against β1- or against β3-integrin. In C, D, neutrophils were untreated, or pre-treated with antibodies against β1-, β2- and β3-integrin, or with a combination of non-specific IgG with matching isotypes. In C, ANOVA showed a significant effect of treatment (p<0.01 in each case); ** = p<0.01 by Dunnett test compared to untreated. In B, D, ANOVA showed significant effect of time but not treatment (p<0.01 in each case); individually, no treatment showed a significant effect on depth of penetration even for the four combined time points. Data are mean ± SEM from 4 experiments (A,B) or three experiments (C,D).

The above results indicated that while β2-integrins were required for neutrophils to adhere to endothelium, they were not required for migration by neutrophils after they had undergone transmigration. We this investigated whether other integrins that were up regulated after transmigration played a role. Blockade of β1- or β3-integrins did not change neutrophil adhesion to the EC (Fig. 7A) or the proportion of neutrophils that migrated initially across the monolayer; transmigration was 90 ± 15% or 102 ± 1% of control for blockade of β1- or β3-integrins respectively (mean ± SEM from 3 experiments). These finding agree with our previous studies [10,11]. However, here for the first time we found that the depth of penetration of the gel by neutrophils was not modified when these integrins were blocked (Fig. 7B). We also pre-treated neutrophils with antibodies against β1-, β2- and β3-integrins combined. Initial adhesion was greatly reduced (Fig. 7C) but those cells that adhered, migrated as far into gels as the untreated controls (Fig. 7D). A combination of isotype matched control antibodies for the three integrins had no effect on adhesion or migration (Fig. 7C,D). Thus, overall, ANOVA for combined time points showed no effect of any antibody or combination of antibodies on depth of penetration.

**Fig 8:**
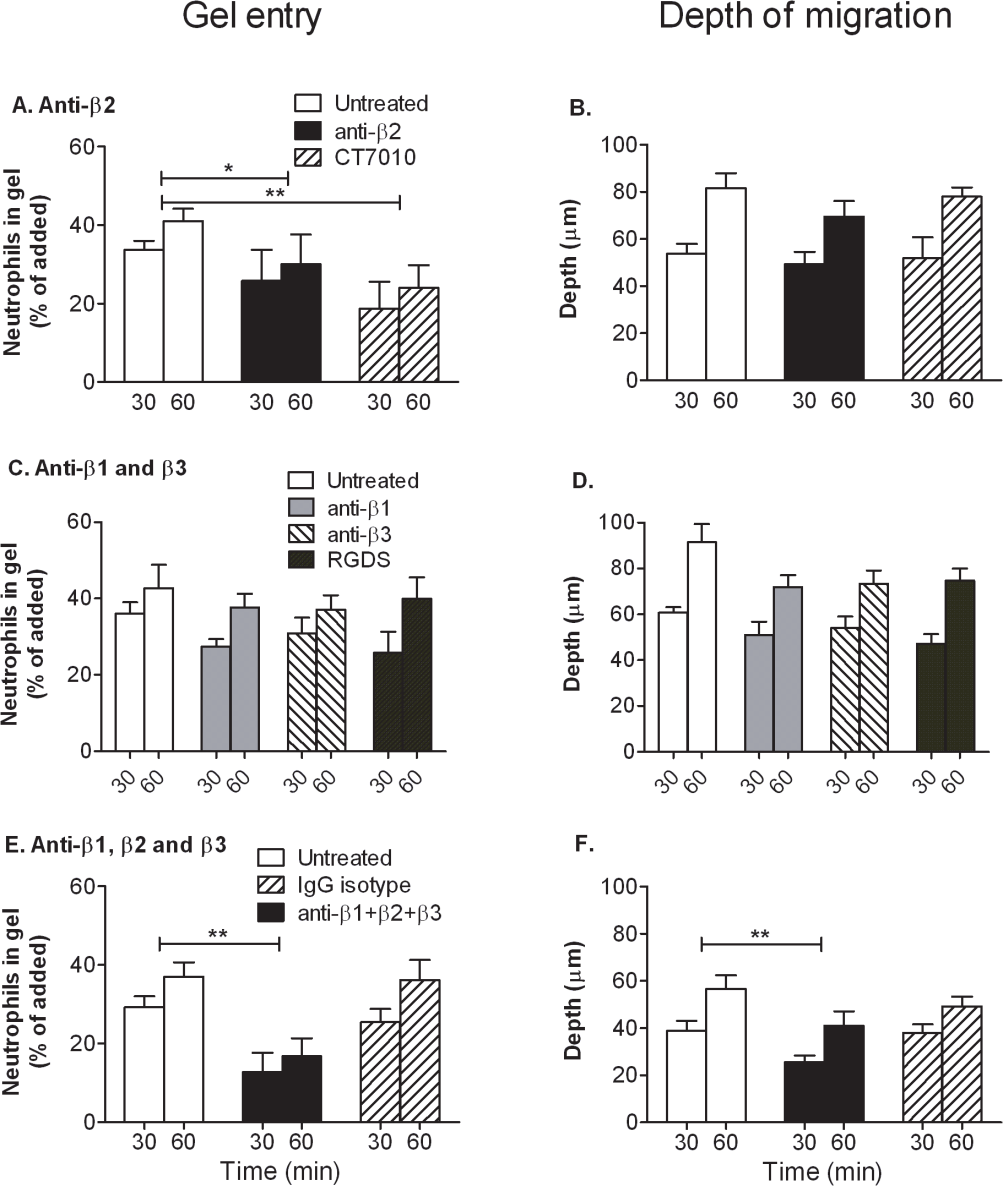
Effects of inhibition of integrin function on entry of neutrophils into collagen gels and depth of penetration induced by fMLP. Collagen gels were formed and loaded with (A) or confocal microscopy (B). B. Representative images of neutrophil nuclei (i) or intracellular bioparticles at 60min (ii) analysed by confocal microscopy., before neutrophils were added and allowed to settle and migrate for 30 and 60 min. A,C,E: Proportion of added neutrophils entering the gel. B,D,F: Average depth penetrated by the neutrophils that had entered. In A, B, neutrophils were untreated, or treated with antibody against β2-integrin or a small molecule inhibitor (CT7010). In C, D, neutrophils were untreated, or treated with antibody against β1 or against β3-integrin, or with RGDS peptide. In E, F, neutrophils were untreated, or pre-treated with antibodies against β1-, β2- and β3-integrin, or with a combination of non-specific IgG with matching isotypes. In A and E, ANOVA showed a significant effect of treatment on entry into the gel (p< 0.01 in each case); in addition, Dunnett test for combined time points showed significant effects of antibodies against integrin or CT7010 (* = p<0.05; ** = p<0.01). In F, ANOVA showed a significant effect of treatment on depth of penetration (p<0.01); in addition, Dunnett test for combined time points showed a significant effect of the combination of antibodies against integrins (* = p<0.01). Data are mean ± SEM from 4 experiments, except for CT7010 tested on 3 occasions.

We next questioned whether ability to migrate independent of integrins was specific to cells that had crossed endothelium, or whether freshly isolated would also migrate in gels independent of integrin engagement. Previous studies by ourselves and others have shown adhesion and migration of freshly-isolated neutrophils on collagen coated surfaces and in some cases, within gels, that was integrin-dependent [23,43,44]. We thus also tested the effect of integrin blockade for freshly-isolated neutrophils migrating directly into gels under the action of the chemoattractant fMLP. When neutrophils were allowed to settle on the gels, fMLP induced ∼40% to enter the gel and these cells reached an average depth ∼50μm by 30min and ∼80μm by 60min (Fig. 8). Blocking β2-integrin function using antibody or CT7010 significantly reduced the proportion of neutrophils entering the gel, but not the depth to which they penetrated, compared to the untreated neutrophils (Fig. 8A,B). Blocking β1- or β3-integrins, or addition of RGDS peptide tended to reduce the number and depth of neutrophils entering the gel slightly, but not significantly (Fig. 8C,D). Combined antibodies against β1-, β2- and β3-integrins reduced entry markedly and reduced depth slightly (about 20%) but significantly, while a combination of isotype matched control antibodies had no effect (Fig. 8E,F). Thus, overall, ANOVA for combined time points only showed an effect when antibodies for β1-, β2- and β3-integrins were used together. If no fMLP was added to the gel, only 1.2 ± 1.2% entered after 60min and those few cells penetrated 9.9 ± 9.8μm (mean ± SEM, n = 3 experiments).

In separate experiments, we tested the effect of antibody against β2-integrin or CT7010 on initial adhesion to the gels rather than penetration. Neutrophils were added and non-adherent cells were washed off after 10min settling. We found that the percentage of neutrophils that adhered fell from 9.3 ± 0.2% for controls to 5.4 ± 0.7% with antibody blockade, a reduction in attachment of 43 ± 5% (mean ± SEM from 3 experiments; p<0.05 by paired t test); in presence of CT7010, adhesion fell from 14.3 ± 1.1% to 3.4 ± 0.7% a reduction of 76±5% (mean ± SEM from 3 experiments; p<0.05 by paired t test). These reductions are greater than the reduction in proportion of cells entering the gels caused by the same agents, suggesting that while the reduction in entry mainly arose from loss of attachment, loss of adhesion did not forbid entry completely. Thus β2-integrins helped support initial attachment and thus entry of gels by freshly-isolated cells, but were not essential for the latter step or for migration once in the gel.

In summary, for freshly isolated neutrophils there was a small effect on depth of penetration when all integrins were blocked, but this was not the case for neutrophils that had migrated through an endothelial monolayer. Thus, the repertoire of integrins used by freshly isolated and transmigrated cells may differ slightly, although in neither case do integrins play a major role in migration within gels.

### Roles of β2-integrins in neutrophil survival or phagocytosis

The foregoing showed that 24h after migration through endothelium, β2-integrin expression was reduced but that migration was independent of this receptor. However, β2-integrins have also been implicated in regulation of neutrophil survival [32,45] and of phagocytosis [27]. This raised the questions whether low levels of apoptosis or decrease in phagocytosis after 24h were influenced by levels of integrin expression. We thus tested whether antibody blockade of β2-integrins would affect apoptosis in the transendothelial migration assay. There was no affect on apoptosis after 24h; 2±1% of antibody-treated cells had condensed nuclei, compared to 3±1% for untreated neutrophils (mean ± SEM from 4 experiments). We also tested whether the specific assay of phagocytosis used in this study (uptake of E. coli bio-particles) required β2-integrin function. Indeed, pre-treatment of freshly isolated neutrophils with mAb against β2-integrin caused reduction in phagocytosis; MFI at 60min fell from 74.5 ± 17.6 for controls to 19.5 ± 6.2 with antibody blockade (mean ± SEM from 6 experiments; p<0.01 by paired t test). Thus, loss of integrin expression and phagocytic capability may have been linked for cells in the gel for a prolonged period.

## Discussion

The responses of neutrophils have been widely studied, both to understand their protective functions, and what may go wrong in inflammatory pathology. Such studies almost invariably use cells freshly-isolated from the blood. This may be appropriate when evaluating initial adhesion to endothelium and transendothelial migration, but subsequent motility, oxidant production and phagocytosis in the tissue are likely to be influenced by changes in the responses of the neutrophils arising from signals during diapedesis. Changes in adhesive and migratory functions immediately after transendothelial migration are well documented [11,12,17] as is the prolongation of survival of migrated neutrophils [28,32]. However, lack of amenable models where adhesion, migration and effector functions of neutrophils can be followed over a prolonged period, means that there is a paucity of information about development of the tissue-resident phenotype. Here, we developed methods to investigate recruitment of neutrophils through endothelium in a 3-D model of tissue, and to follow changes in their function for up to 24 h. Migrated cells were protected from apoptosis and remained motile. Their oxidant production was initially potentiated and then maintained over 24 h, but there was loss of phagocytic capability by 24 h. Interestingly, while changes in integrin expression occurred at mRNA and protein levels, integrin-mediated adhesion was not required for migration in the matrix once the endothelium had been crossed.

A key feature of the model allowing the extended studies was prolonged survival within the matrix after transendothelial migration. While there is some dispute as to how long neutrophils survive in the peripheral circulation before either apoptosis or migration into tissues [46,47], it is evident that they rapidly undergo apoptosis when isolated from whole blood and cultured in standard media on plastic. Neutrophils recovered from body fluids [28], or freshly isolated neutrophils treated with a variety of activators or cytokines including GM-CSF [28,30], or retrieved after migration [48] all showed a degree of protection against apoptosis. We previously collected neutrophils that had migrated through EC on filters and found them to undergo apoptosis much more slowly than non-migrated cells when both were adhered on plastic [32]. It has been shown previously that adherence on protein-coated plastic may itself accelerate apoptosis while adhesion to cytokine-treated endothelial cells may delay it [49]. In none of the above was prolongation of survival comparable with that demonstrated here. Nuclear morphology was well preserved, there was little evidence of caspase 3 activation and the great majority of neutrophils were still motile after 24h in the gel. Even at 48h, less than half appeared apoptotic. When comparing migrated cells to those cultured on plastic, several factors may prolong survival for the former, while adhesion to plastic may have an opposite effect. Interestingly, apoptosis was also suppressed compared to plastic for cells cultured on unstimulated EC. Thus comparatively long survival seen in our model may arise from avoidance of the effects of plastic as well as positive effects from migration and contact with EC. Here, some TNF would remain in cultures even after rinsing, and although it can promote apoptosis under some conditions [50], we previously found that acting alone TNF had little effect on 24h survival [32]. Overall, signals from adhesive interactions and from a cocktail of chemokines and growth factors continuously released into the matrix, whose concentrations would increase for more highly stimulated EC, are likely to have prolonged survival here, as *in vivo*.

Once a model with prolonged survival was developed, we were able to study how neutrophil function developed, particularly in relation to integrin adhesion molecules. Gene expression changed gradually. Total mRNA was constant for several hours, but had halved by 24h. This suggest that the cells become less transcriptionally active by then, although it is also possible that rate of mRNA degradation had increased. After taking this into account, expression of β1- and β3-integrins still tended to increase at mRNA levels, with surface expression increasing by 30–40% by 24h. mRNA for β2-integrin decreased steadily, while surface expression was decreased at all times. It appears that while neutrophils can actively regulate gene expression for many hours after migration, with some increasing and others decreasing, the net effect is to contain less mRNA. The links between the changes in integrin expression and the observed changes in neutrophil behaviour are complex. As expected from past studies [7,8,10,11,51], function-blocking antibody or small molecule inhibitor of β2-integrins greatly reduced adhesion and initial migration through EC, while blockade of β1- or β3-integrins had no effect. However inhibition of each or all of the β1-, β2- or β3-integrins did not modify migration in the subendothelial matrix.

There have been a number of studies of neutrophil migration in collagen gels in vitro, and some in interstitial tissue. Werr and colleagues found that blockade of β1-integrins using antibodies reduced migration velocity of neutrophils in rat intersitium after transendothelial migration induced by superfusing platelet-activating-factor [18,23]. β1-integrin blockade also reduced neutrophil penetration of a collagen gel induced by formylated peptide, fMLP. On the other hand, granulocytes derived from bone marrow of genetically-modified mice that lacked expression of all β1- β2- and β3-integrins did not show impaired migration within gels [24]. In the same studies, dendritic cells injected into skin migrated equally well without integrins. Saltzman et al. [52] found that blockade of β2-integrins did not affect motility induced by fMLP in collagen gels made at concentration similar to that used here, but did reduce motility in much more dilute gels. Koenderman et al. [53] found that neutrophils migrated equally well in fibrin gels with or without blockade of β2-integrins or addition of EDTA to inhibit all integrin function. These above *in vitro* studies of migration in collagen gels did not use cells which had passed through endothelium. In addition, we and others have found that β1- β2- or β3-integrins can all contribute to adhesion of freshly-isolated neutrophils on deposited proteins including collagen and matrix laid down by EC (e.g., [43,44]). Thus, we also used the same blocking agents on freshly-isolated neutrophils migrating into a collagen gel containing chemoattractant fMLP, without an endothelial coating. In this case, we found that β2-integrins supported adhesion to the collagen gel, but had no detectable effect on migration once in the gel. Each integrin sub-family alone had little effect on depth migrated once in the gel, although there was a slight (∼20%) reduction in average depth of penetration when all were blocked. Other studies have suggested that after transendothelial migration, neutrophils differ in integrin usage [11–13]. Here it seems that transmigrated neutrophils do not utilise integrins to migrate in collagen gel. Freshly isolated cells may have some ability to utilise integrins, but even then they can migrate effectively when all are blocked. There is a caveat regarding the differences between transmigrated and non-migrated cells, in that migration after crossing the endothelial monolayer was likely induced by several agents including e.g., CXC-chemokines [8], as well as TNF that was retained in the gel, as compared to fMLP alone in the uncoated gels. This might also contribute to differences in integrin usage. In either case, we have shown for the first time that human neutrophils do not need integrins to move through a collagen matrix once they have crossed endothelium.

We are not aware of studies of the development of effector functions of neutrophils after migration, although one might at least expect initial priming during diapedesis e.g., by cytokines [54]. Even in healthy individuals, neutrophils migrated into lung fluid were in an 'activated' state, with elevated CD11b/α_m_-integrin and ICAM-1 expression [55], which is similar to the phenotype developed in the gel here. Functionally, we found that neutrophils retrieved after 1h had a tendency toward increased basal production of oxidants, but showed marked increase in ability to generate oxidants upon stimulation compared to freshly-isolated cells. After 24h, stimulated oxidant production was still at a level comparable to freshly isolated cells judged by intracellular dye conversion (attributable to H_2_O_2_) or measured by SOD-inhibitable reduction of ferricytochrome C (attributable to superoxide). Phagocytic capability was not evidently primed, being similar to fresh cells for migrated neutrophils retrieved after 1h, but much reduced after 24h. The phagocytosis assay used un-opsonised particles and should therefore be independent of Fc-receptors. It was chosen as being likely to be dependent on β2-integrin expression [27]. Indeed, for freshly isolated neutrophils, blockade of CD18 reduced bacterial uptake by nearly 75%. This suggests that reduced β2-integrins expression after transmigration may have contributed to loss of phagocytosis with time, although reduction in β2-integrins only reached about 35%. Concurrent up regulation of expression of β1- and β3- integrins did not evidently compensate, and in this study, no evident utility for these integrins was found. The loss of phagocytic function may also have arisen from some depression of other cellular responses required for uptake. Even though at 24h apoptosis was negligible, total mRNA was much reduced, suggesting a more generalised reduction in cell activity.

Another interesting aspect of neutrophil responses after migration was that they depended on the degree to which the EC had been stimulated. We previously showed that adhesion and efficiency of transmigration of flowing neutrophils on endothelial monolayers increased with increasing dose of TNF [8,33], but the effect of dose on migration in matrix below EC has not been studied before. Here, not only did accumulation of neutrophils in the matrix increased with dose of TNF, but neutrophils also penetrated further into the gel the higher the dose. Neutrophils recruited at higher TNF dose not only survived better, but also had greater ability to generate oxidants. As noted above, some TNF will be retained in the collagen gel, albeit at lower concentration than that initially added to stimulate the EC due to the various medium changes. While this is unlikely to be the basis of increased survival, TNF is a known activator of neutrophils, so that it may contribute to the changes in their depth of penetration and oxidant production linked to increasing endothelial stimulation. Such pleotropic agents are also likely to be present in inflamed tissue in vivo, and thus our results suggest that upon greater inflammatory insult, not only will the number of neutrophils recruited be greater, but the recruited cells will be more effective.

It was notable that some migrated neutrophils did not disperse in the gel but stayed just beneath the endothelial layer, remaining visible as spread phase-dark cells. These cells gradually reverse migrated onto the top of the EC, and were washed off by rinsing at the different time points, so that cells not in the matrix were nearly all cleared from the system by 24h. We first described the process of reverse migration for neutrophils cultured with EC on a solid dish [42]. The phenomenon has since been observed in mouse models of inflammatory pathology, where the reverse-migrated cells were linked to complications in the lungs arising after ischaemia and reperfusion [56]. Elevation of expression of ICAM-1 was a marker for these cells, and here we found marked up regulation of ICAM-1 mRNA and a progressive increase in surface expression of protein, which had doubled by 1h and increased about five-fold by 24h. This may represent an early movement to the surface from an intracellular store, followed by continuing generation of new protein and surface presentation over time. The functional significance is unclear. ICAM-1 has been shown to support neutrophil-neutrophil interaction [57]. Others have shown specific cytokines, such as GM-CSF can induce neutrophils to express MHC II as well as co-stimulatory molecules such as ICAM-1, which may impart ability to present antigen to T-cells [58]. It might thus be part of a development on adaptive immune function in long-term recruited neutrophils.

In conclusion, using a novel 3-D in vitro model, we were able to investigate the behaviour of neutrophils over prolonged periods after transendothelial migration, and in particular, the changes in integrin expression and function. While expression of different integrins changed in different directions, these adhesion molecules were not required for motility in the sub-endothelial matrix. Adhesion via β2-integrins was required for phagocytosis but not to support the markedly prolonged survival we observed. The degree of 'inflammation' applied to EC influenced development of neutrophil responses, which might also depend on the type or combination of stimuli applied. Thus, the model described could be used to follow and dissect responses of neutrophils in a variety of physiological or pathological scenarios.

## Supporting Information

S1 Video Z-stack recorded 1 hour after neutrophils were added to endothelial cells that had been treated with 100U/ml TNF. Digitised images show neutrophils and endothelial cells at the surface, and then show neutrophils coming into focus as the microscope focuses down through the gel in 2μm steps.
(AVI)

S2 Video Digitised images of neutrophils migrating in a collagen gel 24 hours after migrating through EC treated with 100U/ml TNF. Neutrophils were added and allowed to adhere for 10min before non-adherent cells were washed off and images recorded 24 h later. Phase contrast microscopy was used in a central region of the gel, with images recorded every minute for 10 min. Cells present in the gel above or below the focal plane appear as out of focus.
(AVI)

S3 Video Digitised images of neutrophils migrating in a collagen gel 48 hours after migrating through EC treated with 100U/ml TNF. Neutrophils were added and allowed to adhere for 10min before non-adherent cells were washed off and images recorded 48 h later. Phase contrast microscopy was used in a central region of the gel, with images recorded every minute for 10 min. Cells are present in the gel above or below the focal plane appear as out of focus.
(AVI)
